# Babies Living Safe and Smokefree (BLiSS) Intervention Reduces Children’s Tobacco Smoke Exposure Directly and Indirectly by Improving Maternal Smokers’ Urge Management Skills and Exposure Protection Behaviors

**DOI:** 10.3390/ijerph22020254

**Published:** 2025-02-11

**Authors:** Stephen J. Lepore, Bradley N. Collins, Brian L. Egleston

**Affiliations:** 1 Department of Social & Behavioral Sciences, College of Public Health, Temple University, 1301 Cecil B. Moore Avenue, 9th Floor Ritter Annex, Philadelphia, PA 19122, USA; collinsb@temple.edu; 2 Biostatistics and Bioinformatics Facility, Fox Chase Cancer Center, Temple University Health System, 333 Cottman Avenue, Philadelphia, PA 19111, USA; brian.egleston@fccc.edu

**Keywords:** tobacco smoke exposure, pediatric, tobacco control, smoking cessation, intervention

## Abstract

Children’s tobacco smoke exposure (CTSE) is a public health concern, particularly in low-income and minority communities. Interventions to reduce CTSE have had modest success, and so research must identify mechanisms to improve intervention efficacy. This study investigated mediators of CTSE reduction in an intervention designed to facilitate CTSE protection and maternal smoking abstinence. We analyzed data from the Babies Living Safe and Smokefree (BLiSS) trial, which evaluated the efficacy of a multilevel behavioral smoking intervention initiated in community clinics serving low-income mothers. We estimated direct and indirect effects to evaluate the role of two mediators of the intervention on CTSE at post-intervention follow-up: mothers’ skills in managing smoking urges and their protective behaviors to shield children from TSE. CTSE was measured using mothers’ reports and child cotinine (a CTSE biomarker). The BLiSS intervention was linked to statistically significant lower longitudinal reported CTSE directly and indirectly by increasing mothers’ urge management skills and CTSE protection behaviors (*p*-values < 0.05). The intervention was not directly linked to child cotinine. However, evidence of a statistically significant indirect effect (*p*-value = 0.028) suggested that the intervention reduced longitudinal child cotinine levels by increasing CTSE protection behaviors. Two non-program factors, nicotine dependence and total smokers in the home, also increased child cotinine and reported CTSE (*p*-values < 0.001). Interventions that improve maternal smokers’ urge management skills and CTSE protections can mitigate CTSE. In addition, it is essential to target barriers to CTSE reduction, such as nicotine dependence levels and the presence of other smokers in the home.

## 1. Introduction

Maternal smoking and child tobacco smoke exposure (CTSE) pose well-known public health risks [1]. In the United States, the burden of CTSE is particularly high among children who are black, low-income, and living in rental properties [2,3]. Interventions designed to reduce children’s exposure to parental tobacco smoke have had modest success [4,5], and we have little insight into the mechanisms driving successful CTSE reduction interventions [6]. Moreover, within low-income populations, evidence-based smoking cessation interventions are not highly effective [7,8]. To inform the development of future interventions addressing maternal smoking and associated CTSE in a high-risk population, the present study investigated putative behavioral mediators of an evidence-based smoking intervention, Babies Living Safe and Smokefree (BLiSS). BLiSS was designed to facilitate low-income maternal smokers’ efforts to quit smoking and reduce CTSE [9].

The BLiSS project reached a high-risk population of children exposed to tobacco smoke by partnering with the Philadelphia Special Supplemental Nutrition Program for Women, Infants, and Children (WIC). WIC is a federally funded program that provides access to food, health care referrals, and nutrition education to women with children under six years old who reside in predominantly low-income and minority communities. Trained WIC nutrition counselors delivered a brief tobacco intervention in clinics [10], consisting of Ask (do you smoke?), Advise (about the harms of CTSE), and Refer (to BLiSS trial for cessation services), or “AAR”, to mothers during routine WIC clinic visits. Eligible maternal smokers were then randomized to one of two additional intervention conditions delivered via telehealth by certified tobacco treatment specialists: (1) an attention control intervention (AAR + control) or (2) our novel multimodal behavioral intervention (AAR + MBI). This multilevel approach was designed to educate, support, and build skills and motivation to reduce CTSE and, consequently, promote abstinence skills training. Relative to AAR + control, the AAR + MBI intervention resulted in significantly higher rates of bioverified smoking abstinence among mothers and significantly lower rates of reported CTSE [9].

A behavioral ecological model [11] provided the conceptual framework for BLiSS and guided the identification of hypothesized mediators of intervention effects on CTSE reduction. The model identifies and addresses multiple levels of influence on maternal smoking behaviors, such as social, biological, and cognitive factors, and is grounded in social and cognitive learning theories. While sustained abstinence was the ultimate intervention goal, facilitating mothers’ efforts to reduce CTSE from all sources was essential to achieving this end. Thus, this paper aimed to identify potential mechanisms or mediators accounting for the association between the BLISS intervention and CTSE reduction.

Hypothesized mediators included two critical skills targeted in the intervention: mothers’ smoking urge management and their use of behaviors that protect children from TSE. Urge management skills training is an established component of cognitive behavioral therapy (CBT) treatments for smoking cessation [12]. In the BLiSS intervention, urge management skills training emphasized escaping or avoiding situations that increased smoking urges and delaying smoking when children were nearby while practicing coping strategies, such as cognitive or behavioral distractions, and substituting smoking with other rewarding activities. We paired urge management training with skills training focused on additional behaviors mothers could use to protect children from TSE, including shielding their children from others’ smoking and working with other residents to create a smokefree home. Via telehealth, certified tobacco treatment specialists educated mothers about CTSE harms and the myriad ways children could be exposed (e.g., transfer from the clothing of a smoker, exposure to secondhand smoke in the home) to motivate them to learn and apply behavioral skills that would reduce CTSE. The specialist also engaged in collaborative goal setting and problem solving with mothers regarding reducing CTSE and praised them for implementing CTSE protections. Finally, mothers signed behavioral contracts committing to protective measures to guard against CTSE.

This study evaluated whether the BLiSS intervention effectively improved maternal smokers’ skills in urge management and protecting their children from CTSE and, as a result, reduced CTSE. We hypothesized that the CTSE outcome at post-intervention follow-up would be lower in the AAR + MBI condition than in the AAR + control condition due to greater urge management and CTSE protection behaviors. In addition to testing putative intervention mediators, we explored the effects of non-program factors on CTSE, including maternal nicotine dependence levels, maternal alcohol drinking problems, and the number of smokers in the home. Prior research suggests that these factors may increase children’s risk of TSE, but the findings are mixed and warrant additional research [13,14,15].

## 2. Materials and Methods

### 2.1. Procedures

We analyzed BLiSS trial data (*N* = 396) to test two putative intervention mediators. BLiSS was a two-group (AAR + MBI vs. AAR + control) randomized controlled trial with three data collection waves: pre-intervention baseline (Time 1; T1), 3-month (end-of-treatment) follow-up (Time 2; T2), and 12-month follow-up (Time 3; T3). The self-report data for this analysis were collected via structured, computer-assisted telephone interviews conducted by project staff. For cotinine assays, child urine samples were provided by mothers, who followed standardized protocols for collecting and storing samples. Previous publications describe the study protocol, sample, and setting in detail (e.g., [9]). The Temple University Institutional Review Board approved all procedures, and participants provided informed consent.

Briefly, low-income maternal smokers (70.7% African American; 15.2% Latina) were recruited from Philadelphia, Pennsylvania, WIC clinics. Eligible mothers were current WIC recipients (eligible children <6 years old), current smokers, English speaking, >17 years old, smartphone owners, not pregnant, and able to provide informed consent. All mothers received AAR from WIC nutrition counselors during routine clinic visits, along with self-help print materials about addressing smoking and CTSE (i.e., known health risks, tips for creating a smoke-free home and car, state Quitline number, and information on how to acquire free nicotine replacement therapy [NRT]). WIC staff referred mothers who screened positive for smoking to BLiSS trial staff, who contacted mothers by phone to explain the study, verify eligibility, and obtain informed consent. Eligible, consenting participants completed the T1 data collection interview by telephone before being randomized to condition (199 in AAR + MBI, 197 in AAR + control).

As the name implies, participants in the AAR + MBI experimental condition received the AAR WIC clinic intervention plus the multimodal behavioral intervention. The additional behavioral intervention focused on CTSE reduction, smoking cessation, and relapse prevention. MBI components included up to 5 telephone counseling sessions with a certified tobacco treatment specialist/counselor, integrated adjunctive elements (i.e., mobile app, text messaging, NRT, texted video clips illustrating skills training strategies for TSE reduction, participant binder with worksheets, and the “BLiSS Family Guide” to adopting a smokefree home and protecting children from TSE), and print materials designed to facilitate smokefree home policies (e.g., no smoking signs suitable for posting in the home, family contracts to support no smoking policy adoption and adherence). The MBI intervention content and processes were based on CBT strategies for children’s TSE reduction and maternal smoking cessation. Primary intervention strategies related to CTSE reduction included education about CTSE harms and sources, guidance on specific behavioral strategies to reduce CTSE, including strategies for overcoming social barriers to smokefree home adoption and child TSE protections, and skill training in managing smoking urges and abstaining from smoking. CBT elements included collaborative goal setting, behavioral contracts, problem-solving, and self-monitoring, with counselor feedback facilitated by a web-enabled counselor dashboard linked to the BLiSS mobile app. Participants in the AAR + control condition received the AAR WIC clinic intervention plus a nutrition-focused telephone education intervention, with modes of treatment (e.g., print material, text messages, mobile app) and contact time parallel to the AAR + MBI arm.

### 2.2. Measures

#### 2.2.1. Longitudinal Dependent Measures: Mother-Reported CTSE and Child Cotinine

The primary outcome of the mediation analysis was CTSE, which was assessed using a parent report and a biomarker. Parent observational reports and biomarkers, such as child urinary cotinine, are commonly used to assess CTSE [16]. Including mothers’ observations and child urinary cotinine measurements to assess CTSE leverages the unique strengths of each method. Mothers’ observations can focus on secondhand exposure to tobacco smoke and offer contextual information about the sources (e.g., who, where, and when), which is helpful for understanding patterns and behaviors. However, maternal observations may be inaccurate if children are periodically in others’ care or other locations with smokers and may be subject to recall or social desirability biases. In contrast, urinary cotinine provides an objective, quantifiable biomarker of recent nicotine exposure, free from self-reporting biases. However, it lacks contextual detail and is influenced by other environmental nicotine sources beyond observable secondhand smoke (e.g., contaminated surfaces or thirdhand smoke). Combining these two measurement approaches enhances validity by cross-verifying subjective and objective data.

At T2 and T3, a structured timeline follow-back interview was used to obtain mothers reports of CTSE from all sources (self, other smokers) in the prior seven days. As previously reported, children in the AAR + MBI arm of the trial had lower mother-reported TSE from all sources than children in the AAR + control arm [9]. To improve recall accuracy, we defined exposure for mothers and prompted them to estimate child exposure in different locations and sources [17]. Mothers first recounted how many cigarettes they smoked on weekdays and weekends in the home, in the car, and outside in the previous seven days. The mother then reported how many smoked cigarettes their child was exposed to during these occasions. For in-home smoking, the child just had to be in the home and not necessarily in the same room. Next, parents reported their child’s exposure to cigarettes smoked by others who resided in their home, visited their home, or were encountered outside of their home. The reported CTSE variable was created by summing the number of smoked cigarettes the child was exposed to across all times and sources in the prior seven days (range = 0 to 33.86 cigarettes at T3). To normalize the distribution of the reported CTSE variable for analyses, we applied a square root transformation (normalized range = 0 to 5.80 at T3). We opted for the normalization function based on the histogram of the reported CTSE variable examined before analyzing the outcome.

As previously reported, child cotinine was not significantly different in the experimental versus the comparison group in the BLiSS trial [9]. This lack of difference could be due to various reasons (e.g., cotinine could be affected by thirdhand smoke exposure, which tends to be elevated in low-income residential units—even where residents do not smoke indoors [18]). Nonetheless, we explored whether the intervention indirectly affected child cotinine via the putative mediators. We assayed urine cotinine using validated high-performance liquid chromatography with tandem high-resolution mass spectrometry procedures (0.1 ng/mL limit of quantitation). Child cotinine was not normally distributed and had extreme outliers. Therefore, we winsorized 20 values that exceeded 3 SDs (winsorized range = 0.10 to 738 ng/mL at T3) and log-transformed values before conducting inferential analyses (log-normalized range = −1.00 to 2.90 at T3) [9].

#### 2.2.2. T2 Mediators: Smoking Urge Management Coping Skills and CTSE Protection

Smoking urge management was assessed with the 12-item Tobacco Urge Management Scale (TUMS) [19]. TUMS measures the frequency (1 = never to 4 = often) with which participants applied specific coping skills to manage urges (e.g., “I mentally distracted myself from my craving [for example, meditated, prayed, or listened to music]”, “I escaped situations that made me crave a cigarette—I left high-risk situations”, and “I substituted smoking with something else, like food or sugarless candy when I felt an urge to smoke”). Items were summed (possible range = 12–48), with higher scores indicating the more frequent use of urge management coping skills. The measure had good internal consistency (McDonald’s Omega = 0.87). CTSE protection was assessed using the 11-item Parent Reported Exposure Protection Scale (PREPS) [20]. PREPS measures the frequency (1 = never to 4 = often) with which participants engaged in behaviors to reduce or eliminate their child’s TSE (e.g., “ask people to not smoke around child”, “move child away from others who are smoking”, “post ‘no smoking’ signs around home”). Items were summed (possible range = 11 to 44), with higher scores indicating more frequent CTSE protective behaviors. The measure had adequate internal consistency (Cronbach’s alpha = 0.71).

#### 2.2.3. T1 Non-Program Predictors: Nicotine Dependence Levels, Alcohol Drinking Problems, and Other Smokers in the Home

The nicotine dependence level of participants was assessed with a single item from the Fagerstrom Test for Nicotine Dependence (FTND): “How soon after waking up do you smoke your first cigarette?” [21]. This single item correlates highly with the total FTND score (*r* = 0.717) [22]. Alcohol drinking problem (yes/no) was assessed with the Tolerance Worry Eye-opener Amnesia K/cut down (TWEAK) measure [23]. Finally, participants reported each person who lived in their home and whether they smoked. This question determined the number of other smokers in the household.

### 2.3. Analytic Approach

For descriptive purposes, we used Pearson’s *r* correlations to show the degree and direction of the relationships among the variables. Then, in parallel analyses, we examined the effect of randomization on longitudinal reported CTSE and cotinine outcomes, respectively. The primary analyses used traditional random effects linear regressions with a random intercept for participant. We entered as covariates into the regressions the study wave (i.e., T2 versus T1 and T3, with T2 as the reference) as categorical main effects terms and as interaction terms with the experimental condition variable. We included the interaction terms to assess possible longitudinal changes in the intervention effect. However, the interaction test did not find statistically significant treatment effect differences between T2 and T3 outcomes. Therefore, we focused on the main effect of intervention and the non-program factors on reported CTSE and child cotinine. Three potentially confounding non-program factors—the number of smokers in the home, the participant’s level of nicotine dependence, and alcohol use problems—were also included in the models. To examine mediation effects, we began with a traditional multi-mediator path analysis using direct adjustment [24]. As a sensitivity analysis, we also used a statistical causal inference approach to estimate mediation pathways separately for urge coping and TSE protection efforts [25,26]. Unlike traditional methods, the causal inference model estimates population-level effects rather than conditional effects. Specifically, this model calculates the mediators’ natural direct and indirect effects [25] and uses bootstrap standard errors to test the significance of the indirect effects [26]. In an additional sensitivity analysis, we examined whether adding a random intercept for site and participant improved the fit of the models. However, adding the site-specific intercept did not significantly improve the models (*p*-values > 0.49 by likelihood ratio tests). Thus, we only accounted for repeated measures within participants in each model. We used multiple imputations with 25 imputed datasets for the path and natural effects analyses to account for missing data [27]. A *p*-value < 0.05 was the criterion for statistical significance. We used SAS version 9.4 (SAS Institute, Cary, NC, USA) to impute datasets and STATA version 15 (StatCorp, College Station, TX, USA) for all other analyses.

## 3. Results

### 3.1. Zero-Order Correlations

Table 1 shows Pearson’s correlations among the study variables. As predicted, mothers’ levels of urge management and CTSE protective behaviors were significantly and negatively correlated with reported CTSE. Similarly, protective behaviors were significantly and negatively correlated with child cotinine. However, mothers’ levels of urge management were unrelated to cotinine. Of the non-program factors, mothers’ nicotine dependence and the number of household smokers were significantly and positively correlated with reported child TSE and cotinine. In addition, mothers’ nicotine dependence was significantly and negatively correlated with their TSE protective behavior. The level of alcohol problems was not associated with any variable.

### 3.2. Mediation Analyses: Reported CTSE and Child Cotinine Outcomes

We first report the traditional modeling results for each outcome, followed by the sensitivity analysis using causal modeling techniques. All models control for nicotine dependence, the number of household smokers, and alcohol problems at T1.

**Figure 1:**
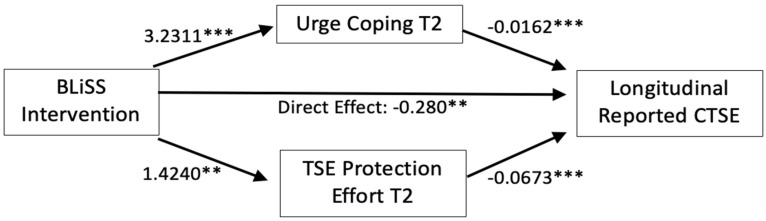
Hypothesized meditation path model testing direct and indirect effects of BLiSS intervention participation on longitudinal reported child tobacco smoke exposure. Notes: T2 = 3-month end-of-treatment follow-up; CTSE = child tobacco smoke exposure. ** *p*-value ≤ 0.01, *** *p*-value ≤ 0.001 two-tailed.

Table 2 and Figure 1 show the longitudinal reported CTSE model results. The coefficients show the effect of a one-unit change in the covariates on the outcomes (the square root of reported CTSE or the mediators). The total effect on the reported CTSE was statistically significant. As hypothesized, the paths between the intervention and the mediators, the intervention and the outcome, and the mediators and the outcome were statistically significant, and in the predicted directions. Two non-program factors, nicotine dependence and the number of household smokers, were also significantly and positively related to reported CTSE, which was consistent with the correlation analysis. Importantly, Table 3 also shows that the indirect effects of the intervention on longitudinal reported CTSE via the putative mediating variables were statistically significant. As shown in Figure 1, being randomized to the AAR + MBI condition was associated with using more urge management coping skills and engaging in more reported CTSE protective behaviors at T2. Each of these behaviors, in turn, was associated with a lower level of longitudinal reported CTSE.

As shown in Table 2, the total effect of the intervention on normalized longitudinal reported CTSE was −0.4276 (standard error [SE] 0.123, *p* = 0.001). Of this, 12.2% of the intervention effect could be explained by the urge coping pathway, 22.4% was due to the reported CTSE protection behavior effect pathway, and the remaining variance in reported CTSE was due to the intervention’s direct effect.

The results of the causal inference model aligned with those of the traditional model. The natural total effect was −0.4276 (SE 0.123, *p* = 0.001). The natural direct effect of the intervention independent of urge coping on reported CTSE was −0.3395 (SE 0.120, *p* = 0.005), and the natural indirect effect was −0.0882 (SE 0.031, *p* = 0.004). The natural direct effect of the intervention independent of protection behavior was −0.3200 (SE 0.116, *p* = 0.006), and the natural indirect effect through the protection behavior pathway was −0.1076 (SE 0.043, *p* = 0.013).

**Figure 2:**
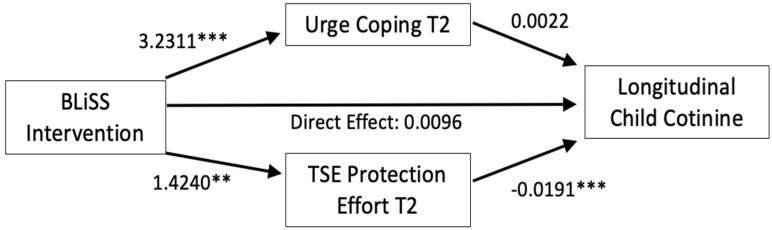
Hypothesized meditation path model, testing direct and indirect effects of BLiSS intervention participation on longitudinal winsorized log cotinine. Notes: T2 = 3-month end-of-treatment follow-up. ** *p*-value ≤ 0.01; *** *p*-value ≤ 0.001 two-tailed.

Table 3 and Figure 2 show the longitudinal child cotinine model results. The coefficients show the effect of a one-unit change in the covariates on the outcomes (winsorized log cotinine outcome or the mediators). The direct and total effects of the intervention on cotinine were not statistically significant, and there was no evidence of an indirect effect on cotinine via urge coping. However, there was evidence that the intervention indirectly affected longitudinal cotinine via mothers’ CTSE protection efforts at T2. As shown in Figure 1, the intervention was associated with more CTSE protective behavior by mothers, which, in turn, was associated with lower child cotinine levels. Finally, two non-program factors, nicotine dependence and the number of household smokers, were significantly and positively related to cotinine, which was consistent with the correlation analyses (see Table 3).

The results of the causal inference model aligned with the traditional model. The natural total effect was −0.0106 (SE 0.059, *p* = 0.857). The natural direct effect of the intervention independent of urge coping on winsorized log cotinine was −0.0075 (SE 0.060, *p* = 0.900), and the natural indirect effect through the cotinine pathway was −0.0031 (SE 0.010, *p*= 0.748). The natural direct effect of the intervention, independent of protection behavior, was 0.0151 (SE 0.060, *p* = 0.801), and the natural indirect effect through the protection behavior pathway was −0.0257 (SE 0.012, *p*= 0.028).

## 4. Discussion

Among a high-risk population of maternal smokers from predominantly low-income and minority communities, a multilevel intervention (MBI + AAR) initiated in urban community WIC clinics attenuated reported CTSE at follow-up, directly and indirectly, by increasing mothers’ use of smoking urge management skills and adoption of protective behaviors to minimize their children’s exposure. Mothers’ protective behaviors accounted for relatively more of the intervention benefits on reported CTSE outcomes than their urge management behaviors. Further, the intervention indirectly affected child cotinine at follow-up via mothers’ protective behaviors. The analyses also identified non-program risk factors that require further attention in future interventions, specifically addressing nicotine dependence and the number of smokers in the household. The traditional and causal model results aligned, suggesting that the findings are robust to modeling assumptions.

There is considerable evidence that coping with urges is essential for smoking cessation and relapse prevention [28,29]. However, this may be the first study to show the significance of guiding smokers’ efforts with urge management strategies that extend to CTSE reduction goals. The finding that protective behaviors had a more substantial role in mediating the effects of the intervention on CTSE may be explained by the facts that (a) protective behaviors extend beyond the mothers’ smoking behaviors to other sources of exposure (e.g., prohibiting in-home smoking, moving children away from others who smoke) and (b) protective behaviors include methods of reducing exposure, even when mothers succumb to urges and smoke (e.g., mother smokes outside the home or changes clothing after smoking). Given the evidence, we recommend both treatment elements be included in cessation interventions targeting smokers with children.

The significant and positive associations between CTSE and two non-program factors—nicotine dependence and the number of household smokers—point to additional targets of intervention. It is possible that behavioral urge management skills may be most effective at helping smokers with relatively low nicotine dependence levels curb their smoking around children. Among those with higher dependence, it may be necessary to add additional evidence-based intervention components, such as NRT [30]. Evidence suggests that relative to single formulations, combination NRT therapies (e.g., nicotine patch + gum or lozenge) are effective at controlling withdrawal and cravings [31]. Pediatric office-initiated interventions targeting parents who smoke to reduce CTSE sometimes include NRT [e.g., 14]. Regarding other smokers, interventions could include skill building to help smokers marshal support and cooperation from other smokers. Although greater awareness of secondhand smoke’s harms can promote protective behaviors at home [32], our findings suggest that in households with multiple smokers, increasing maternal smokers’ knowledge alone is insufficient to protect children. Additional intervention components may be necessary, such as helping maternal smokers to address barriers to changing other smokers’ behaviors (e.g., identifying alternative smoking locations and removing household smoking paraphernalia) [33] and developing negotiation skills through role-playing.

### 4.1. Strengths

Our use of data from a randomized controlled trial to identify mediators of a smoking intervention in a high-risk population is a strength. Other strengths include longitudinal modeling, sensitivity analyses, and the use of multiple imputations to address missing data. A final strength is the inclusion of potential confounders in the models and the identification of non-program factors that could influence CTSE.

### 4.2. Weaknesses

Despite the study’s strengths, a notable limitation is that the two measures of CTSE produced slightly different results. Using mothers’ reports of CTSE, we observed the direct effects of the intervention on CTSE and indirect effects through both mediators. In contrast, using child cotinine to measure CTSE, we only found an indirect effect of the intervention on CTSE through mothers’ protective behaviors. These divergent effects of the intervention on mother-reported CTSE versus on the cotinine biomarker are consistent with the literature [34]. The correlation analyses (Table 1) showed that the observational and biomarker measures of CTSE are significantly and positively correlated, but modestly so. It is tempting to conclude that biomarkers are more reliable CTSE measures because they are less subjective. However, that would mostly be true for understanding children’s level of overall nicotine exposure as opposed to exposure to secondhand tobacco smoke, as observed by the mothers. For example, there is evidence that children who live in homes with an indoor smoking ban or who live in impoverished communities and multi-family housing units can have elevated cotinine levels [35]. This evidence points to the pervasiveness of thirdhand tobacco smoke exposure as well as secondhand smoke from adjoining smokers’ residences in low-income communities, e.g., [18]. Children’s cotinine levels in the BLiSS trial were likely affected by these various exposure sources, given that many families studied lived in persistently impoverished communities with primarily old, poorly maintained apartments that have housed generations of smokers. In these circumstances, it is possible to eliminate indoor household smoking and still find elevated biomarkers of tobacco exposure. Thus, our cotinine data may not fully reflect the degree of participants’ efforts to reduce observable CTSE. Another explanation for the divergent intervention effects on reported CTSE versus cotinine is that the measures reflect different amounts of exposure. The self-report measure captures exposure over a week, whereas the cotinine measure captures recent exposure (e.g., 2–3 days). Also, a one-time measure of cotinine may not be sensitive enough to capture intervention effects (for a full discussion of the limits of cotinine, see [34]). The null direct effect of the intervention on child cotinine reduction suggests the ongoing need to enhance the potency of strategies designed to reduce CTSE. For example, parents’ biomarker monitoring of CTSE could improve their exposure risk awareness [36] and motivation to protect their children beyond the most easily observed indoor exposures.

## 5. Conclusions

This study underscores the value of incorporating skills training in urge management and CTSE protective behaviors into smoking cessation interventions aimed at maternal smokers whose children are at risk for CTSE. By aligning urge management training with practical strategies to minimize CTSE from all sources, interventions can potentially address both direct and indirect sources of CTSE. The findings also point to the importance of addressing non-program factors, such as nicotine dependence and the presence of multiple smokers in the household, through complementary components like NRT and skill-building for negotiating household smoking behaviors. Future research should explore the role of combined NRT and enhanced urge management skills training to support sustained reductions in CTSE, particularly for highly dependent smokers and other vulnerable groups. Given the chronic nature of nicotine addiction, innovative and adaptable intervention strategies will be essential to reducing CTSE effectively and sustainably in households where multiple smokers are present.

## Figures and Tables

**Table 1:** Pearson’s correlations among study variables using pooled multiple imputed data set.

Variable	1	2	3	4	5	6
1. Urge coping T2						
2. TSE protection effort T2	0.317 ***					
3. Nicotine dependence T1	−0.077	−0.132 **				
4. Household smokers T1	−0.088	−0.046	0.091			
5. Alcohol problems T1	−0.036	−0.029	−0.015	−0.061		
6. Reported CTSE T3	−0.286 ***	−0.339 ***	0.160 **	0.255 ***	−0.030	
7. Child cotinine T3	−0.035	−0.114 *	0.172 ***	0.137 **	−0.052	0.368 ***

Notes: T1 = pre-intervention baseline; T2 = 3-month end-of-treatment follow-up; T3 = 12-month follow-up; CTSE = child tobacco smoke exposure. * *p*-value ≤ 0.05, ** *p*-value ≤ 0.01, *** *p*-value ≤ 0.001 two-tailed.

**Table 2:** Results from adjusted path analysis on longitudinal square root normalized child tobacco smoke exposure reported by mothers.

Path	Estimate (*SE*)	*p*-Value
*Direct effect of intervention on outcome* Intervention → longitudinal child TSE	−0.2796 (0.115)	0.015
*Effects of intervention on mediators*		
Intervention → T2 urge coping	3.2311 (0.858)	<0.001
Intervention → T2 TSE protection effort	1.4240 (0.501)	0.004
*Effects of mediators on outcome*		
T2 Urge coping → longitudinal child TSE	−0.0162 (0.006)	0.006
T2 TSE protection effort → longitudinal child TSE	−0.0673 (0.011)	<0.001
*Indirect effects of intervention on outcome*		
Intervention × T2 urge coping	−0.0522 (0.024)	0.031
Intervention × T2 TSE protection effort	−0.0958 (0.037)	0.011
*Total effect*	−0.4276 (0.123)	0.001
*Multivariable direct effect of control variables on outcome*		
T1 Nicotine dependence → longitudinal child TSE	0.1668 (0.044)	<0.001
T1 Household smokers → longitudinal child TSE	0.5334 (0.092)	<0.001
T1 Alcohol problems → longitudinal child TSE	0.0343 (0.123)	0.780

Notes: Both mediators and potential confounders were included in the multivariable analysis. T1 = pre-intervention baseline; T2 = 3-month end-of-treatment follow-up; TSE = tobacco smoke exposure.

**Table 3:** Results from adjusted path analysis on longitudinal winsorized log cotinine level.

Path	Estimate (*SE*)	*p*-Value
*Direct effect of intervention on outcome* Intervention → longitudinal cotinine	0.0096 (0.061)	0.875
*Effects of intervention on mediators*		
Intervention → T2 urge coping	3.2311 (0.858)	<0.001
Intervention → T2 TSE protection effort	1.4240 (0.501)	0.004
*Effects of mediators on outcome*		
T2 Urge coping → longitudinal cotinine	0.0022 (0.003)	0.477
T2 TSE protection effort → longitudinal cotinine	−0.0191 (0.005)	<0.001
*Indirect effects of intervention on outcome*		
Intervention x T2 urge coping	0.0071 (0.010)	0.494
Intervention x T2 TSE protection effort	−0.0273 (0.012)	0.026
*Total effect*	−0.0107 (0.060)	0.859
*Multivariable direct effect of control variables on outcome*		
T1 Nicotine dependence → longitudinal cotinine	0.1056 (0.025)	<0.001
T1 Household smokers → longitudinal cotinine	0.2041 (0.050)	<0.001
T1 Alcohol problems → longitudinal cotinine	0.0274 (0.082)	0.738

Notes. Both mediators and potential confounders were included in the multivariable analysis. T1 = pre-intervention baseline; T2 = 3-month end-of-treatment follow-up.

## Data Availability

The data are not publicly accessible for privacy and ethical reasons. However, the data used for this analysis are available through the corresponding author upon reasonable request.

## Abbreviations

AAR: Ask: Advise, Refer; BLiSS: Babies Living Safe and Smokefree; CBT: cognitive behavioral therapy; CTSE: child tobacco smoke exposure; MBI: multimodal behavioral intervention; NRT: nicotine replacement therapy; TSE: tobacco smoke exposure; WIC: Special Supplemental Nutrition Program for Women, Infants, and Children; T1: Time 1; T2: Time 2; T3: Time 3.
